# Supplementary material for: A chemostat-based model for growing bacterial biofilms
Source: Microbiol Spectr. 2025 Oct 27;13(12):e02333-25. doi: 10.1128/spectrum.02333-25 (PMC12671209; doi:10.1128/spectrum.02333-25)
Supplement: Supplemental material — Supplemental methods and results. [file spectrum.02333-25-s0001.pdf]

Supplemental Information for

A Chemostat-Based Model for Growing Bacterial Biofilms

Fabrizio Spagnolo, Iñigo Caballero, Alexandra Goldblatt, Michael J. Loccisano, Mazharul I. Mahe, Yaxkyn Mejia, Naya Melvani, Aliza Nagel, Aiden Stanciu, Sherin Kannoly, John J. Dennehy, & Monica Trujillo

Methods

Experiments relating to the characterization of FS11

Visualization of ancestral and chemostat-evolved strain

The ancestral strain of MPAO1, JJD122 (our lab designation for the HER #1369 strain sourced from the Felix d'Herelle Center at the Université Laval), and the chemostat-evolved strain described in Methods, FS11, were grown exponentially (OD=0.4) in LB media. After washing the strains with sterile phosphate buffered saline (PBS), 8 µl of 4% paraformaldehyde (PFA) was added to 8 µl of each of the cultures and incubated at room temperature for 30 minutes to fix the sample. For transmission electron microscopy (TEM), the fixed samples were applied to 200-mesh copper grids (Ted Pella, Redding, CA) and incubated at room temperature for 10 minutes. Excess liquid was blotted away using Thermo Scientific Western Blotting Filter Paper, and a 4% paraformaldehyde solution was used to fix cells. The samples were then stained with 2% uranyl acetate, blotted again, and left to air-dry. Electron micrographs were captured using a JEOL (Akishima, Tokyo, Japan) JEM 1400-Flash with an AMT (Woburn, MA) NS15 camera.

Growth Curves for the FS11 strain

Creating growth curves for the experimental strains proved difficult. The strains of *P. aeruginosa* used have a phenotype whereby planktonic cells begin self-aggregating at some point in the exponential growth phase. This aggregation shifted any optical measurement and made data based upon them unreliable. Several methods of generating growth curves were attempted, including automated measurements in 96-, 24-, and 6-well plates in a plate reader with various amounts of shaking; manual readings using a sidearm flask and a manual optical density reader and even a fluorescence-based approach was attempted. All failed to yield reliable results. For these reasons, no growth curve or μ_{max} are reported here.

Experiments Relating to the Visualization of Biofilms

Visualizing Biofilms via phase-contrast Microscopy

Studies of planktonic and biofilm populations were conducted using the FS11 strain. Brightfield microscopy images of planktonic and homogenized biofilms samples were taken using a Nikon (Tokyo, Japan) Ti2-E Inverted Microscope System. 50 µl of each sample was aliquoted into 35 mm MatTek (Ashland, Massachusetts) glass bottom dishes. Images were captured using the 100x objective under oil immersion and were processed using Nikon's NIS elements platform version 5.42.01.

Visualizing Biofilms via fluorescent Microscopy

Studies of biofilm architecture and size, along with cellular activity, were conducted using the JJD529 GFP-marked strain. CTC (5-Cyano-2,3-ditolyl tetrazolium chloride from Biotum) was used to evaluate the respiratory activity of biofilms. Healthy cells respiring via electron transport chain will absorb and reduce CTC into an insoluble, red fluorescent product. A 10 mg/mL stock

solution was diluted to 1 mg/mL with Davis media. DAPI NucBlue™ Fixed Cell ReadyProbes™ Reagent (DAPI) was used to evaluate total number of cells. 10 µl of DAPI and 200 µl of CTC solution were aliquoted and mixed in the glass bottom dish. The biofilm coverslip was placed in the middle of the dish avoiding bubble formation by gently pushing the coverslip to the bottom with tweezers. Afte 10 minutes incubation the staining solution was washed off by washing twice with sterile PBS. We took images of the biofilm-associated cells using a Nikon Eclipse Ti-2 microscope (Tokyo, Japan) with Nikon LEDI laser units and a Hamamatsu ORCA-FusionBT digital camera (model C15540, Shizuoka, Japan). Images were captured and processed using Nikon NIS-Elements AR software. Images were taking according to the manufacturer's instructions to capture the CTC, DAPI and GFP fluorescence.

Control Experiments Relating to Quantification of biofilms

Quantification of Homogenized versus Homogenized and Sonicated biofilms.

These experiments were conducted using the JJD529 GFP-marked strain. Secondary biofilm sampling was done as described in Methods and filtered through a 70 µm cell strainer (Avantor/VWR, Cat. No. 76327-100) in order to minimize chances of clogging the 100 µm sorting chip nozzle. The total volume (10 mL) was divided into two 5 mL fractions. The experimental fraction was sonicated on ice at the frequency 20 kHz (amplitude: 25) for a total of 2 minutes, alternating between 5 seconds on and 5 seconds off, using a QSonica Q55 sonicator and 1/8" probe (QSonica, Newtown, CT). The sonicated 5 mL as well as non-sonicated control 5 mL sample were then serially diluted as described under methods. After dilution, we plated 100 µL from each sample in triplicate. Following incubation, the CFU/mL were automatically counted using the SphereFlash colony counter.

Evaluation of biofilm treatments effects via FACS

These experiments were conducted using the JJD529 GFP-marked strain. We used fluorescence-activated cell sorting (FACS) to evaluate the effects of homogenizing or homogenizing and sonicating biofilms. In all cases, the FACS utilized was a SONY SH800S Cell Sorter (SONY Biotechnology, San Jose, CA) equipped with 488 nm & 561 nm Lasers and FL1 and FL3 filter sets. Sorting runs were completed with a sorting chip with a 100 µm nozzle size. Secondary biofilms were processed as described above. A total volume of 1 mL of each of the samples was analyzed by flow cytometry. In addition, we used 1 mL of filtered Davis media as a negative control to calibrate the FACS machine. The excitation wavelength used to detect GFP fluorescence was 488 nm.

Results

Control Experiments Relating to the Visualization of Biofilms

Visualizing Biofilms via phase-contrast Microscopy

Phase-contrast and fluorescent microscopy was used to visualize several biofilm samples. A key insight of microscopy-based analysis was the presence of floating aggregates, conglomerations of cells bound together that were not part of the primary or secondary biofilms (Fig S1, yellow arrows). The source and/or role of these floating aggregates is unknown but may be important in the development of secondary biofilms or in the transition of planktonic cells into biofilm-associated phenotypes. Additionally, the visual comparison of the images corresponding to

planktonic and homogenized biofilms suggest our homogenization protocol successfully yields mostly single cells.

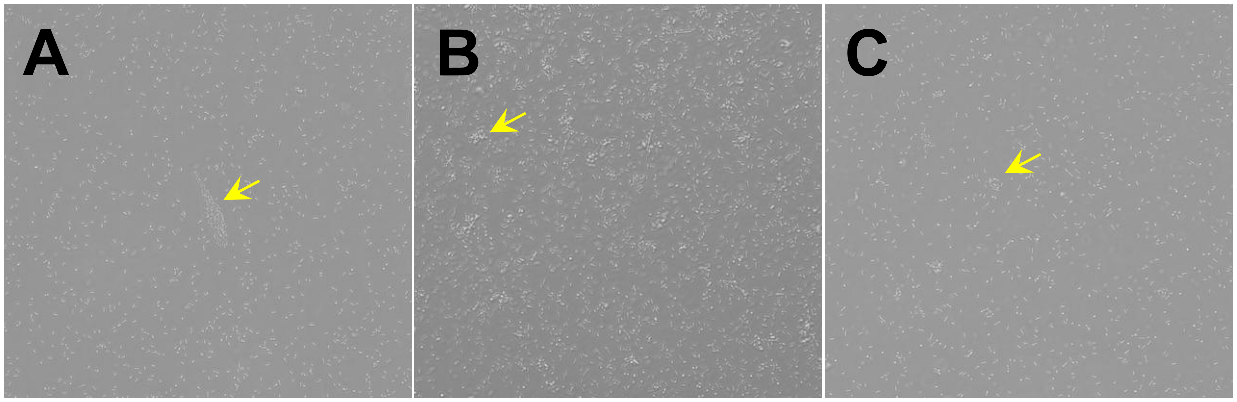

Figure S1: Brightfield Microscopy of different cell populations. A) Planktonic cells. B) Homogenized biofilm from cover slip. C) Homogenized biofilm from wall. All images were taken using a Nikon (Tokyo, Japan) Ti2-E Inverted Microscope System. Images were captured using the 100x objective under oil immersion and were processed using Nikon's NIS elements platform.

Visualizing Biofilms via fluorescent Microscopy

These experiments, conducted with the JJD529 GFP-marked strain helped to control for the possibility of counting large numbers of dead or metabolically inactive biofilm-associated cells in the FACS experiments. Figure 3 show the results of fluorescent microscopy. Analysis of the images suggests that most of the cells are alive.

Control Experiments Relating to Quantification of Biofilm

Comparisons viable cells count with different treatments of biofilms

One potential source of experimental error in biofilm studies is quantification of biofilm population sizes (1–5). Measuring the population size of individual cells within a biofilm may be problematic because the nature of biofilms is for cells to remain aggregated, rather than solitary.

If the cells are still aggregated viable counts may under-estimate population sizes because colonies that were started by aggregates are assumed to have been founded by single cells.

Homogenizaton of the biofilms as described in Methods and serial dilution with replicated plating was used as baseline. To test the possibility that homogenization was not complete, after homogenization the sample was divided in two. One half was sonicated, and the control was not. Viable cells were counted for both the non-sonicated and sonicated sample using serial dilution with replicated plating. In the data collected, the means of the CFU/mL for the secondary biofilm population samples under the two conditions, sonication after homogenization or only homogenization was calculated (Fig S2), they are not significantly different (one-tailed t-Test p=0.14).

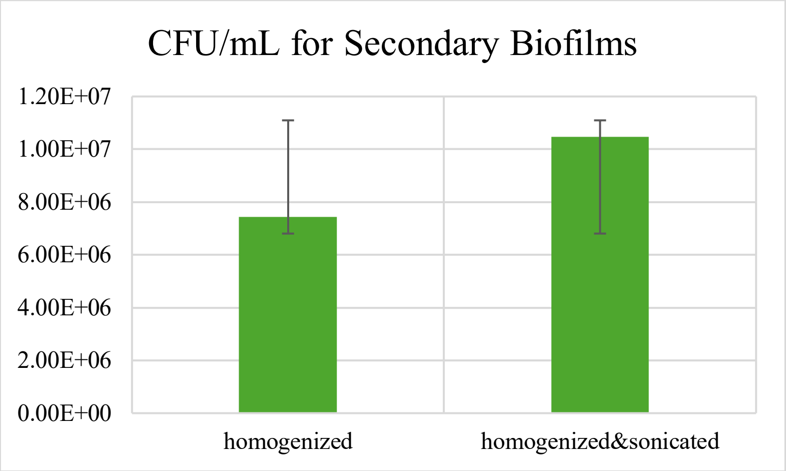

Figure S2: Mean of triplicate plating experiments for secondary biofilm after homogenization vs homogenization and sonication.

FACS analysis of biofilms samples undergoing different treatments.

Analysis of the FACS results (Fig S3) suggests that the biofilm homogenization protocol developed is similarly effective as sonication followed by plating or sonication followed by flow cytometry.

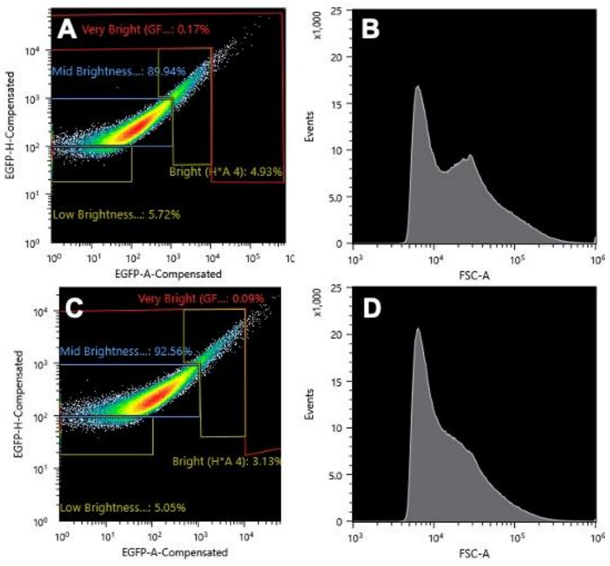

Figure S3: FACS-based counting of Homogenized vs Homogenized & Sonicated Samples. A) Homogenized samples were counted using the SONY SH800S Cell Sorter until 1 million events were recorded. Investigator-designated gates for the counts are indicated. The gold colored "bright" gate indicates that 4.93% of the million events are in this area. B) A histogram of the events reported in Panel A. The second, small peak at the FSC-A of $3x10^4$ indicates that there are suspected multi-cell aggregates in the homogenized sample. C) Homogenized and sonicated sample results, with gates indicated. The gold-colored gate indicates that 3.13% of the million events are in this area. D) Histogram of events reported in Panel C. Note the absence of a secondary peak, indicating that the extra step of sonicating did decrease the number of suspected aggregates from 4.93 to 3.13%, a measurable but not statistically significant difference.

Dilution	μL/min	*D*	Planktonic Generation Time (hr)
Low	140.6	0.28	2.5
High	333.3	0.67	1

Table S1: Dilution Rates. The dilution rates described in the text as either "Low" or "High" are described. Dilution (*D*) in chemostats is the rate of addition of new media relative to the working volume of that chemostat. The working volume of our chemostats is 30 mL. The physiological effect of *D* on planktonically growing cells within a chemostat is to control the expected generation (doubling) time of bacterial cells. For more details on chemostat theory, see (6).

Homoserine Lactone	Experimental Concentration 2:1 (ng/L)	Experimental Concentration 4:1 (ng/L)
N-butyryl-L-homoserine lactone	400	400
N-(3-oxododecanoyl)-L-homoserine lactone	200	100

Table S2: Homoserine Lactone Concentrations. Experiments with homoserine lactones (HSLs) had HSLs added at ratios of either 2:1 or 4:1. Final experimental concentrations are listed here. For sources and preparation of the HSLs, see the main text.

REFERENCES CITED

1. Peeters E, Nelis HJ, Coenye T. 2008. Comparison of multiple methods for quantification of microbial biofilms grown in microtiter plates. J Microbiol Methods 72:157–165.

2. Bakke R, Kommedal R, Kalvenes S. 2001. Quantification of biofilm accumulation by an optical approach. J Microbiol Methods 44:13–26.

3. Prieto B, Silva B, Lantes O. 2004. Biofilm quantification on stone surfaces: comparison of various methods. Sci Total Environ 333:1–7.

4. Wilson C, Lukowicz R, Merchant S, Valquier-Flynn H, Caballero J, Sandoval J, Okuom M, Huber C, Brooks TD, Wilson E, Clement B, Wentworth CD, Holmes AE. 2017. Quantitative and Qualitative Assessment Methods for Biofilm Growth: A Mini-review. Res Rev J Eng Technol 6:1–42.

5. Malone M, Bjarnsholt T, McBain AJ, James GA, Stoodley P, Leaper D, Tachi M, Schultz G, Swanson T, Wolcott RD. 2017. The prevalence of biofilms in chronic wounds: a systematic review and meta-analysis of published data. J Wound Care 26:20–25.

6. Dykhuizen DE. 1993. Chemostats used for studying natural selection and adaptive evolution. Methods Enzymol 224:613–631.
